# Knowledge on and intake of Folic acid supplementation in pregnant women in Denmark

**DOI:** 10.1186/1743-8454-7-S1-S25

**Published:** 2010-12-15

**Authors:** Mikkel Mylius Rasmussen, Dorte Clemmensen, Claus Mosdal

**Affiliations:** 1 Department of Neurosurgery, Aalborg Hospital, Aarhus University Hospital Hobrovej 18-22, Posboks 365, 9100 Aalborg, Denmark; 2 Department of Neurosurgery, Aarhus Hospital, Aarhus University Hospital Nørrebrogade 44, 8000 Århus C, Denmark

## Background

Folic acid (FA) deficiency is related to neural tube defects (NTD). In a non risk pregnancy The Danish National Board of Health recommends a FA supplementation from planned pregnancy until 3 month after conception. We looked into pregnant women’s knowledge about and actual supplementation of FA and related this to education, number of pregnancies and age.

## Materials and methods

Eighty four consecutive pregnant women with a midwife consultation were included from the 25th - 28th of August 2008. All filled out a unified questionnaire.

## Results

82% had knowledge of FA supplementation and 89% had a FA supplementation. 51% followed national recommendations. We found statistically significant correlation between higher educational level and knowledge about FA supplementation, actual supplementation of FA and FA supplementation in correlation with national recommendations. No statistical relations were found between number of pregnancies or age and FA related parameters. Family, friends, general practitioner (GP) and the internet were main information sources.

## Conclusions

Correct FA supplementation is quite low, correspondingly knowledge about and supplementation of FA is fairly high. Further intervention is necessary to increase correct FA supplementation. Women with low educational level, perhaps as an indicator for low socio-economic status seem as a group of interest. Multiple pregnancies or higher age cannot be seen as indicators for a higher information level. Information to the pregnant women incorporating family, friends, GP or the internet is suggested.

